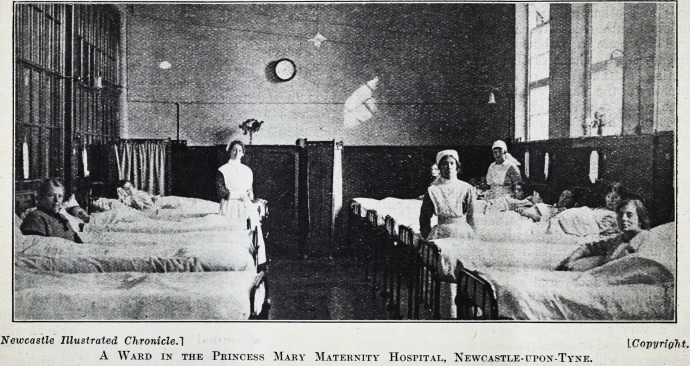# Princess Mary at Newcastle

**Published:** 1924-01

**Authors:** 


### PRINCESS MARY AT NEWCASTLE.

### WHAT A £5 NOTE DID.

NEWCASTLE Maternity Hospital, which Princess Mary visited recently, was founded by Act of Parliament as long ago as 1760. In 1819 Dr. Thomas Elliot, Surgeon to the Hospital, transmitted to the Treasurers of the Charity a donation of £5, with the condition that "it should be vested at common interest with the Corporation of this town, in aid of any future fund which the subscribers and public may be disposed to raise for the purpose of erecting, or purchasing, any appropriate building for a Lying-in Hospital in Newcastle; or in aid of any other means the governors and subscribers may, at any future day, deem necessary to adopt, to carry the proposition, as above-mentioned, into effect." This was the commencement of what is known as "Elliot's Fund," which, in the course of a short time, reached the total of £1,371, and enabled the Governors of the Institution to erect the building as it exists in New Bridge Street, which was opened for the reception of patients in 1826. The number of cases is steadily on the increase, and in 1922, owing to the very limited accommodation of the New Bridge Street building, a house was rented in Higham Place, and the nursing and domestic staff transferred to it, which, however, relieved the over-crowded condition of the hospital only for a very short period.

*Newcastle Illustrated Chronicle.*] [*Copyright.*

A WARD IN THE PRINCESS MARY MATERNITY HOSPITAL, NEWCASTLE-UPON-TYNE.

### A GENEROUS GIFT.

The Committee of Management, having found it impossible to provide further accommodation for the great increase in the number of patients seeking treatment, approached the Newcastle Corporation with reference to the Industrial School site in City Road, which was soon to become vacant. The Corporation, generously appreciating the circumstances, and fully realising the value of the work done by the hospital, purchased the Industrial School site, and gave a lease to the Trustees of the hospital of part of this site for a period of thirty years. Last March the reconstruction of portions of these buildings began, and the Institution was transferred from New Bridge Street to Jubilee Road in September. On the occasion of her visit Princess Mary formally opened the entrance door to the hospital and went over several wards, the theatre and nursery. She also visited the Nurses' Carnival, which was a very successful function and lasted for ten days. Her Royal Highness was presented with a silver rose bowl for herself, and a silver spoon and porringer for her son, and she cut a huge birthday cake, which was given to the patients.

## Figures and Tables

**Figure f1:**